# Notch inhibitors induce diarrhea, hypercrinia and secretory cell metaplasia in the human colon

**DOI:** 10.17179/excli2021-3572

**Published:** 2021-04-26

**Authors:** Michael Collins, Jean-Marie Michot, Christophe Bellanger, Charlotte Mussini, Karim Benhadji, Christophe Massard, Franck Carbonnel

**Affiliations:** 1 Department of Gastroenterology, Kremlin Bicêtre Hospital, Assistance Publique-Hopitaux de Paris, Le Kremlin Bicêtre, France; 2 Paris Sud University, Le Kremlin Bicêtre, France; 3 INSERM, U1193, Paul-Brousse University Hospital, Hepatobiliary Centre, Villejuif, France; University Paris-Sud, Université Paris-Saclay, Faculté de Médecine Le Kremlin-Bicêtre, France; Assistance Publique-Hôpitaux de Paris (AP-HP), Pôle de Biologie Médicale, Paul-Brousse University Hospital, Villejuif, France; 4 Drug Development Department (DITEP), Gustave Roussy, Université Paris-Sud, Université Paris-Saclay, Villejuif, France; 5 Department of Pathology, Kremlin Bicêtre Hospital, Assistance Publique-Hopitaux de Paris, Le Kremlin Bicêtre, France; 6 Eli Lilly and Company, Indianapolis, USA

**Keywords:** notch inhibitor, gamma secretase inhibitor, secretory cell metaplasia

## Abstract

In humans, inhibition of Notch oncogenic signaling leads to tumor regression. Preclinical studies indicate that Notch signaling contributes to the maintenance of intestinal homeostasis. Here, we sought to describe the intestinal effects of a first-in-human Notch inhibitor in an indication of refractory cancer. Between 2014 and 2017, adult patients treated for refractory cancer with the novel Notch inhibitor LY3039478 and who had grade ≥ 2 diarrhea were referred to the gastroenterology department of a tertiary hospital in the Paris region of France. Eleven patients (median (range) age: 72 (29-83)) were included in the study. All patients had advanced cancer: adenoid cystic carcinoma (n=3, 27 %), sarcoma (n=3, 27 %), and other types (n=5, 46 %). In all cases, digestive tract endoscopy revealed abundant mucus in the intestinal lumen, and digestive tract biopsies showed an abnormally low proportion of enterocytes and marked elevation of the proportion of pseudostratified goblet cells. Microscopic inflammation was seen in colon biopsies from 2 of the 11 patients (18 %). The clinical, endoscopic and histological abnormalities were dependent on the dose of Notch inhibitor. All patients resolved their digestive signs or symptoms after discontinuing the dose and the median (range) time interval between discontinuation of the Notch inhibitor and resolution of all the gastrointestinal signs and symptoms was 7 days (4-24). Likewise, the median time interval between discontinuation and resolution of the histological abnormalities was 7 days (1-10). Blocking Notch signaling induces secretory cell metaplasia of the intestinal epithelium, which in turn leads to transient diarrhea. Our results confirm the role of Notch signaling in intestinal homeostasis in humans.

## Introduction

The human intestinal epithelium forms villi, each of which comprises three distinct compartments. The crypt is located at the bottom of the villus, and the intermediate compartment lies between the crypt and the apex (the most differentiated part). Progenitor stem cells and Paneth cells are prominent in the crypt epithelium, whereas the apex contains most of the enterocytes and approximately 10 % of the goblet cells and neuroendocrine cells (Potten, 1998[26]; Karam, 1999[11]; de Santa Barbara et al., 2003[5]). The stem cells differentiate as they migrate from the crypt to the apex. The proportions of absorptive and secretory cells within the villus axis are closely regulated, with a balance between proliferation, apoptosis, differentiation, migration, and adhesion. The main molecular pathways involved in these processes are Wnt, BMP, PTEN/Akt, Hedgehog, and Notch (Scoville et al., 2008[29]). 

Notch signaling pathway is conserved through the eukaryotic reign; it regulates the differentiation of progenitor cells and/or the maintenance of stem cell status. Notch signaling appears to be required for intestinal homeostasis (Fortini, 2009[7]; Fre et al., 2011[8]; Vooijs et al., 2011[33]). Notch ligands (Jagged 1 and 2 and Delta-like ligands 1 and 2) are secreted in a paracrine fashion by the stromal cells and bind to the four isotypes of the Notch receptor. Ligand binding leads to membrane domain cleavage by a gamma secretase complex and thus the release of an intracellular domain. The latter translocates to the nucleus and regulates gene expression.

Alterations in the Notch signaling pathway (mutations in ligands and/or receptors, or the overexpression or impaired translocation of these proteins) have been described in several malignancies, including lymphoid leukemia, melanoma, glioblastoma, and cancers of the breast, ovary, lung, pancreas, colon, head and neck, cervix, and kidney (Radtke and Raj, 2003[28]; Koch and Radtke, 2007[13]; Puente et al., 2011[27]). The Notch activation pathway might also promote an epithelial-to-mesenchymal cell transition, cancer progression, and metastasis (Chanrion et al., 2014[3]). Gamma secretase inhibitors are the mainstay of Notch-related drug therapy (Krop et al., 2012[14]; Lee et al., 2015[15]; Messersmith et al., 2015[20]; Massard et al., 2016[18]). These newly developed drugs are being trialled in hematological cancer (Knoechel et al., 2015[12]; Papayannidis et al., 2015[25]) and solid tumors (Krop et al., 2012[14]; Lee et ayl. 2015[15]; Locatelli et al., 2017[16]; Massard et al., 2018[17]). The main adverse events associated with Notch inhibition affect the digestive tract; diarrhea occurs in up to 73 % of treated patients (Krop et al., 2012[14]; Lee et al., 2015[15]; Papayannidis et al., 2015[25]; Massard et al., 2018[17]; Mir et al., 2018[22]). Hence, a better understanding of these intestinal adverse events might help to optimize the use and safety of notch inhibitors in oncology.

In humans, the underlying mechanisms and the clinical characteristics of the intestinal adverse events induced by gamma secretase inhibitors have yet to be fully characterized. The objectives of the present study were to describe, investigate and better understand intestinal adverse events in patients treated with gamma secretase inhibitors and to explore the Notch pathway's physiological role in intestinal homeostasis.

## Patients and Methods

### Study design and patient selection

This was a retrospective, observational study of a cohort of consecutive adult patients treated with a novel Notch inhibitor (LY3039478, Eli Lilly and Company, Indianapolis, IN, USA) in phase I clinical trials at Gustave Roussy (Villejuif, France) and who were referred for drug-induced diarrhea to the gastroenterology department at Bicêtre University Hospital (Le Kremlin-Bicêtre, France) between October 2014 and October 2017. LY3039478 is a novel, potent, gamma secretase inhibitor that is in clinical development (Massard et al., 2018[17]; Mir et al., 2018[22]; Even et al., 2020[6]). Patients were randomized to increasing oral doses of LY3039478 in three-times-in-a-week (TIW) regimen or to two-week loading dose period and then 50 mg TIW (Clinicaltrials.gov: NCT01695005) (Massard et al., 2018[17]; Mir et al., 2018[22]; Even et al., 2020[6]). Each treatment cycle lasted 28 days. The Notch inhibitor was discontinued in the event of tumor progression or unacceptable toxicity. Patients referred to the gastroenterology department at Bicêtre University Hospital underwent a stool culture, a screen for *Clostridioides difficile* toxin, and flexible sigmoidoscopy with biopsies as part of routine care. Differential diagnoses (intestinal infection or tumor-related symptoms) were ruled out in order to establish a causal relationship between diarrhea and exposure to the gamma secretase inhibitor. The study was approved by an independent ethics committee (CPP Ile de France VII, Paris, France), and referenced in clinicaltrials.gov: NCT01695005 and conducted in accordance with the tenets of the Declaration of Helsinki and its updates.

### Data collection

Clinical, laboratory, endoscopic and histological data were collected retrospectively from medical records. The severity of diarrhea was assessed according to the Common Terminology Criteria for Adverse Events (version 4.03). All drugs received within the three months before initiation of the gamma secretase inhibitor and during the trial were recorded. Colonoscopies or flexible sigmoidoscopies were performed by two gastroenterologists (MC or FC). The endoscopy findings were rated on a semi-quantitative scale: 0 for normal findings, 1 for mild abnormalities, 2 for moderate abnormalities, and 3 for severe abnormalities. All gastrointestinal biopsies were centrally reviewed by a single pathologist (CM). Tissue sections were stained with hematoxylin-eosin-saffron reagent or probed in an immunohistochemical assessment for carcinoembryonic antigen (CEA) antibody and the mucin MUC2.

### Statistical analysis

Quantitative variables were described as the median (range), and qualitative variables were described as the number (percentage). Correlations were tested with a non-parametric Spearman test. All statistical analyses were conducted with R studio software (version 1.1.383).

## Results

Among the 130 patients treated with the novel Notch inhibitor LY3039478 at Gustave Roussy cancer centre (Massard et al., 2018[17]; Mir et al., 2018[22]; Even et al., 2020[6]), 11 were referred to the gastroenterology department at Bicêtre University Hospital and thus were included in the study. The 11 patients had grade 2 diarrhea (n=4) or grade 3 diarrhea (*n*=7). The participants' main clinical characteristics are summarized in Table 1(Tab. 1). The median (range) time interval between Notch inhibitor initiation and the onset of diarrhea was 24 days (7-196). None of the patients reported abdominal pain on presentation. Patients had a median of five liquid stools per day (1-15). The patient who received the highest dose of Notch inhibitor (100 mg TIW) had the most severe diarrhea (15 liquid stools per day) and required intravenous fluids. Patients who received 75 mg TIW (n=5) had 6 (2-6) liquid stools per day. Patients who received 50 mg TIW (n=5) had 3 (1-7) liquid stools per day. Hence, there was a positive correlation between the dose received and the number of stools (rho=0.79, p=0.006). The median (range) time between treatment initiation and onset of diarrhea was 24 (7-196) days and this variable was not correlated with the dose received (rho=-0.35, p=0.29; data not shown).

### Endoscopic findings

**Figure 1:**
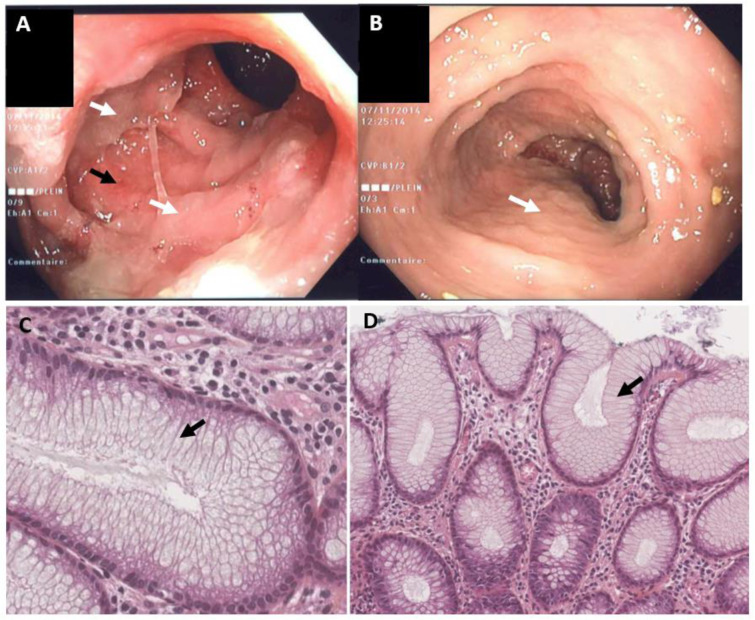
Endoscopic findings (A) and histological findings (B) in a 70-year-old female patient having developed diarrhea following the initiation of treatment with the Notch inhibitor LY3039478 (75 mg TIW). The patient was being treated for metastatic breast cancer. Figures 1A and B show the sigmoid colon during an endoscopy. The lumen of the colon is filled with mucus (white arrows). In Figure 1A (but not Figure 1B), one can see slight erythema/loss of a vascular pattern underneath the mucus (black arrows). Figures 1C and 1D show endoscopic colon biopsies (stained with hematoxylin-eosin-saffron) from the patient in Figures 1A and 1B (magnification: x400 in 1C and x200 in 1D). Both Figures 1C and 1D show the total replacement of enterocytes by goblet cells (arrow), in the absence of changes in architecture or inflammation.

All patients underwent sigmoidoscopy or colonoscopy (Figure 1(Fig. 1)). None of the patients had ulceration or mucosal bleeding. The patient who had received 100 mg TIW had abundant mucus in the lumen of the colon and a thick mucus layer on the mucosa. In the patients who received 75 mg TIW (n=5), abundant mucus in the lumen was also observed, and three also showed erythema and a slight loss of the vascular pattern.

In the patients who had received 50 mg TIW (n=5), mucus in the lumen was either absent (n=2), slightly visible (n=2) or moderately visible (n=1). Mild inflammation (erythema and loss of the vascular pattern) was observed in the rectum of one patient. The patient having received 100 mg TIW and the 5 patients having received 75 mg TIW had abundant mucus in the lumen (grade 3 - severe), whereas patients who had received 50 mg TIW had either no visible mucus (n= 2), slightly visible mucus (n=2), or moderately visible mucus (n=1).

### Histological findings

Endoscopic biopsies of the colon revealed hypercrinia in all the patients. This was characterized by an abnormally low proportion of enterocytes and an abnormally high proportion of pseudostratified goblet cells. In patients who had received 100 mg or 75 mg TIW, all the enterocytes had been replaced by pseudostratified goblet cells (Figure 1C and 1D(Fig. 1)). In patients who had received 50 mg TIW, the hypercrinia was less marked.

Two patients had evidence of microscopic inflammation of the colonic mucosa, including a few cryptic abscesses and mild neutrophil infiltration. One patient had an elevated intraepithelial lymphocyte count (>50 CD3^+^ T cells per 100 enterocytes).

### Outcomes

All patients resolved their digestive signs or symptoms after discontinuing the dose and the median (range) time interval between discontinuation of the Notch inhibitor and resolution of all the gastrointestinal signs and symptoms was 7 days (4-24). The patient who received 100 mg TIW had diarrhea for 8 days. The 5 patients who received 75 mg TIW had diarrhea for 8 days (5-24), and patients who received 50 mg TIW had diarrhea for 5 (4-9) days. There was no correlation between the dose received and duration of diarrhea (Spearman's p=0.1, rho = 0.5). The diarrhea in the patient with an elevated intraepithelial lymphocyte count resolved after four days. Flexible sigmoidoscopy was performed in four patients at a median of 7 (1-10) days after resolution of diarrhea; the findings were normal in all of cases. Biopsies showed a normal brush border, normal enterocytes but persistent, partial hyperplasia of the goblet cells.

One patient with a locally advanced uterine leiomyosarcoma died of tumor progression. Among the six patients who had received 100 mg or 75 mg TIW, four resumed treatment with LY3039478 at a dose of 50 mg TIW. Three of the six did not have diarrhea thereafter. One of the three patients with renewed diarrhea discontinued the study. Three patients received corticosteroids with a view to treating diarrhea but there were no obvious beneficial or harmful effects.

See also the Supplementary data.

## Discussion

The present study is the first to have described the intestinal adverse events associated with Notch inhibition in humans. The clinical/pathological picture was remarkably homogeneous and was specific for this class of drugs. The main adverse event was diarrhea related to hypercrinia, with a dose-dependent decrease in the proportion of enterocytes and marked hyperplasia of goblet cells. Diarrhea and goblet cell metaplasia resolved seven days after treatment discontinuation, which is consistent with the intestinal epithelium's turnover time. In murine models, Notch inhibitors induce goblet cell hyperplasia and the accumulation of mucins in the lumen; full recovery is observed after 5 days (Milano et al., 2004[21]; Wong et al., 2004[34]; van Es et al., 2005[32]). Our observations are in line with these data and emphasize the role of Notch signaling in human intestinal homeostasis.

The mucus layer is an essential component of the intestine's mucosal barrier. It is primarily composed of glycoproteins (such as MUC2) that prevent pathogens from accessing the epithelium. Ulcerative colitis is associated with goblet cell depletion, thinning of the mucus layer, and MUC2 glycosylation defects; these may lead to intestinal inflammation (Van der Sluis et al., 2006[31]; Shan et al., 2013[30]; Johansson et al., 2014[10]). In patients with ulcerative colitis, Notch signaling is activated in the colonic crypts (Okamoto et al., 2009[24]). Correction of this activation might be beneficial in the treatment of ulcerative colitis. However, Notch's functions are complex (Noah and Shroyer 2013[23]). Notch signaling induces M cell differentiation (Hsieh and Lo 2012[9]) and promotes barrier function by lamina propria lymphocytes (Dahan et al., 2011[4]). Moreover, there is evidence to show that blocking Notch signaling aggravates *Citrobacter rodentium*-induced and dextran-sulphate-sodium-induced colitis in mouse models (Okamoto et al., 2009[24]; Ahmed et al., 2012[1]; Mathern et al., 2014[19]). Although Notch pathway inhibition downregulated the expression of pro-inflammatory cytokines, it also diminishes IL-10 secretion and impairs T-reg lymphocyte function. Recent research has shown that blocking the Notch pathway can disrupt the crypt's stem cell compartment and thus leads to dysbiosis and inflammation in a *C. rodentium* rodent model (Ahmed et al., 2018[2]). Furthermore, gamma secretase inhibitors exacerbate experimental colitis, due to loss of the regenerative response within the epithelium. Further studies are needed to investigate the potential therapeutic value of gamma secretase inhibitors in intestinal inflammation (Okamoto et al., 2009[24]). 

Notch inhibitors have shown antitumor activity in phase 1 and 2 trials in patients with relapsed or refractory hematological malignancies or solid tumors. Intestinal adverse events are frequent and appear to be a class effect with gamma secretase inhibitor and is consistent with the inhibition of Notch signaling. We showed that these intestinal adverse events are dose-dependent and recedes rapidly after treatment cessation.

## Conclusion

Gamma secretase inhibitors dose-dependently induce diarrhea, hypercrinia and secretory cell metaplasia, as characterized by an abnormally low proportion of enterocytes and marked hyperplasia of goblet cells. All these changes had reversed one week after treatment cessation. Our findings confirm the Notch pathway's role in intestinal homeostasis.

## Notes

Michael Collins and Jean-Marie Michot contributed equally as first author.

## Disclosures

Dr Jean-Marie Michot discloses to be principal/sub-investigator of clinical trials for: Abbvie, Aduro, Agios, Amgen, Argen-x, Astex, AstraZeneca, Aveo pharmaceuticals, Bayer, Beigene, Blueprint, BMS, Boeringer Ingelheim, Celgene, Chugai, Clovis, Daiichi Sankyo, Debiopharm, Eisai, Eos, Exelixis, Forma, Gamamabs, Genentech, Gortec, GSK, H3 biomedecine, Incyte, Innate Pharma, Janssen, Kura Oncology, Kyowa, Lilly, Loxo, Lysarc, Lytix Biopharma, Medimmune, Menarini, Merus, MSD, Nanobiotix, Nektar Therapeutics, Novartis, Octimet, Oncoethix, Oncopeptides AB, Orion, Pfizer, Pharmamar, Pierre Fabre, Roche, Sanofi, Seattle Genetics, Servier, Sierra Oncology, Taiho, Takeda, Tesaro, Xencor

Pr Christophe Massard discloses to be principal/sub-investigator of clinical trials for: Abbvie, Aduro, Agios, Amgen, Argen-x, Astex, AstraZeneca, Aveo pharmaceuticals, Bayer, Beigene, Blueprint, BMS, Boeringer Ingelheim, Celgene, Chugai, Clovis, Daiichi Sankyo, Debiopharm, Eisai, Eos, Exelixis, Forma, Gamamabs, Genentech, Gortec, GSK, H3 biomedecine, Incyte, Innate Pharma, Janssen, Kura Oncology, Kyowa, Lilly, Loxo, Lysarc, Lytix Biopharma, Medimmune, Menarini, Merus, MSD, Nanobiotix, Nektar Therapeutics, Novartis, Octimet, Oncoethix, Oncopeptides AB, Orion, Pfizer, Pharmamar, Pierre Fabre, Roche, Sanofi, Seattle Genetics, Servier, Sierra Oncology, Taiho, Takeda, Tesaro, Xencor

Dr Karim Benhadji discloses to be employed by Eli Lilly and Company, Indianapolis, USA.

## Funding

NCT01695005 study was funded by Eli Lilly and Company, Indianapolis, USA.

## Non-financial support

Drugs, equipment supplied by the entity, travel paid by the entity, writing assistance, administrative support, etc.: AstraZeneca, Roche, Novartis, Gilead, Celgene, Bristol-Myers Squibb, GSK.

## Authors’ contributions

Study concepts: MC, FC, JMM, KB

Study design: FC, MC

Data acquisition: CB, CM, CM, JMM, MC 

Quality control of data and algorithms: MC, FC

Data analysis and interpretation: MC, FC, JMM

Statistical analysis: MC, CB

Manuscript preparation: JMM, MC 

Manuscript editing: JMM, MC

Manuscript review: all authors.

## Acknowledgements

The authors thank David Fraser PhD (Biotech Communication SARL, Ploudalmézeau, France) for copy-editing assistance.

## Supplementary Material

Supplementary data

## Figures and Tables

**Table 1:**
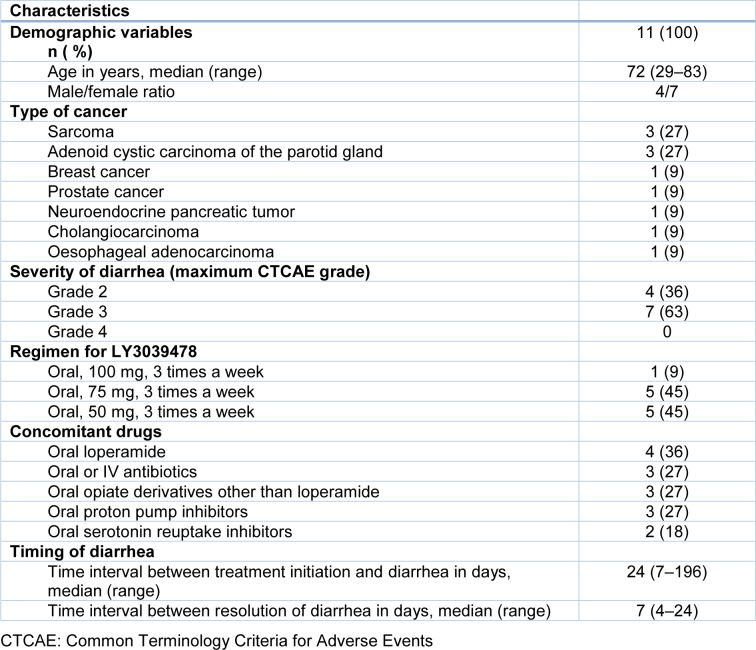
Demographic and clinical characteristics of patients investigated for diarrhea related to Notch inhibitors
